# A long short-term memory network with SHAP interpretability for dynamic prediction of ICU delirium: development and external validation

**DOI:** 10.3389/fdgth.2026.1856311

**Published:** 2026-07-31

**Authors:** Lanqiong Lei, Hongya Xia, Ran Zhang, Yu Huang, Fang He, Shuwen Huang, Xusihong Cai, Juan Yang, Jing Zhou

**Affiliations:** 1 Department of Intensive Care Unit, The Affiliated Hospital of Zunyi Medical University, Zunyi, Guizhou, China; 2 Nursing Department, The Affiliated Hospital of Zunyi Medical University, Zunyi, Guizhou, China; 3 Zunyi Medical University, Zunyi, Guizhou, China; 4 Department of Neurology, The Second Affiliated Hospital of Zunyi Medical University, Zunyi, Guizhou, China; 5 Department of Joint Surgery, Tongren People’s Hospital, Tongren, Guizhou, China; 6 School of Nursing, Zunyi Medical University, Zunyi, Guizhou, China

**Keywords:** delirium, ICU, interpretability, LSTM, prediction model, SHAP

## Abstract

ICU delirium is a common and serious complication in critically ill patients, yet existing prediction models rely predominantly on static data and lack interpretable decision logic, limiting their clinical utility. This retrospective cohort study enrolled 464 ICU patients from the First Affiliated Hospital of Zunyi Medical University as the internal cohort and 70 patients from the Second Affiliated Hospital as an external test set. A variable system comprising nine baseline characteristics and 24 dynamic variables was established through systematic evidence synthesis and a clinical pilot investigation. Dynamic variables were organized into 24-hour rolling observation windows (T1–T7), and a two-layer bidirectional long short-term memory (LSTM) network incorporating a masking mechanism and class-weight correction was developed to predict delirium occurrence in each window based on all preceding sequential inputs. The model achieved an area under the receiver operating characteristic curve (AUCROC) of 0.835 (95% CI: 0.724–0.946), sensitivity of 0.696, and specificity of 0.894 on the internal test set, with satisfactory calibration and clinical net benefit on this set across a broad probability threshold range. External validation yielded an AUCROC of 0.830 (95% CI: 0.716–0.944), with calibration and decision curve analysis in the external cohort further supporting reliable probability estimates and clinical net benefit, indicating stable cross-institutional generalizability. SHapley Additive exPlanations (SHAP) analysis revealed a clinically coherent, phase-dependent transition in dominant predictors: invasive mechanical ventilation predominated during T1–T3 (peak mean |SHAP| = 0.286 at T3), arterial pH and Sequential Organ Failure Assessment (SOFA) score emerged as principal drivers during T4–T6, and SOFA score retained the highest attribution at T7, collectively providing an evidence-based rationale for temporally differentiated monitoring and intervention strategies.

## 1 Introduction

Delirium is an acute neuropsychiatric syndrome characterized by disturbances in attention, awareness, and cognition, with a fluctuating clinical course typically precipitated by acute physiological stressors such as infection, metabolic derangement, and iatrogenic exposure. In the ICU, delirium affects 20%–80% of critically ill patients and is independently associated with prolonged mechanical ventilation and increased in-hospital mortality (1, 2). Cognitive impairment persists beyond 12 months post-discharge in a substantial proportion of survivors (3). Given this clinical burden, early identification of high-risk patients to enable timely preventive intervention represents a critical unmet need in critical care medicine.

Current clinical assessment of ICU delirium relies predominantly on validated bedside instruments, including the Confusion Assessment Method for the ICU (CAM-ICU) and the Intensive Care Delirium Screening Checklist (4). While these tools demonstrate acceptable diagnostic sensitivity and specificity under controlled conditions, their real-world applicability is substantially constrained. Bedside assessment depends on nursing availability and workload, resulting in inconsistent evaluation frequency and systematic gaps during high-acuity periods (5). More fundamentally, these instruments are designed to confirm the presence of established delirium rather than to predict its imminent occurrence. Their static assessment logic cannot detect the pathophysiological signals that precede clinical manifestation. Machine learning-based prediction models have partially addressed this gap, with pooled AUROCs of up to 0.89 reported for ICU delirium prediction in meta-analytic evidence (6). However, most existing models rely on single-time-point data, using a static feature vector to generate risk estimates while discarding the temporal ordering and evolutionary dynamics of the clinical signal (7). Furthermore, the inherent opacity of complex machine learning architectures remains a barrier to clinical adoption, as risk scores without interpretable attribution are insufficient to guide targeted intervention (8).

Long short-term memory (LSTM) networks are a class of recurrent neural networks designed for sequential data modeling. Through gating mechanisms that selectively retain clinically relevant long-range dependencies while filtering short-term noise, LSTM addresses the temporal limitations of static approaches (9). LSTM has demonstrated superior discriminative performance over conventional machine learning methods in multiple time-sensitive critical care prediction tasks, including sepsis, acute kidney injury, and heart failure risk stratification (10–12). These results establish a strong methodological precedent for applying LSTM to ICU delirium prediction. To address the interpretability deficit inherent in deep learning models, SHapley Additive exPlanations (SHAP) offers a principled solution. As a game-theoretic framework that quantifies the marginal contribution of each input feature to individual predictions (13), SHAP can render LSTM decision logic transparent and clinically actionable.

Accordingly, this study aimed to (1) construct a variable system for ICU delirium risk prediction through systematic evidence synthesis and clinical pilot validation; (2) develop and externally validate a bidirectional LSTM model capable of dynamically updating delirium risk estimates across 24-hour rolling observation windows throughout the ICU stay; and (3) apply SHAP-based interpretability analysis to characterize the temporal evolution of feature contributions across observation windows, thereby providing an empirical foundation for stage-specific clinical monitoring and intervention strategies.

## 2 Materials and methods

### 2.1 Study design and participants

This study employed a retrospective cohort design conducted across two tertiary-level ICUs. The internal cohort comprised patients admitted to the ICU of the First Affiliated Hospital of Zunyi Medical University between January 2024 and July 2025. This cohort was partitioned into training, validation, and internal test sets at a 70%:15%:15% ratio using stratified random sampling, with allocation performed at the patient level to preclude data leakage across subsets. The external test set was independently assembled from patients admitted to the ICU of the Second Affiliated Hospital of Zunyi Medical University between January 2025 and August 2025, and was withheld entirely from all stages of model development and hyperparameter optimization.

Patients were eligible for inclusion if they met all of the following criteria: age ≥18 years, ICU length of stay ≥48 h, and availability of at least one valid CAM-ICU assessment during the admission. Patients were excluded if they had a pre-existing diagnosis of dementia, schizophrenia, or other severe psychiatric disorders; if consciousness impairment was attributable to traumatic brain injury, neurosurgical intervention, or primary central nervous system disease precluding delirium assessment; or if severe sensory deficits rendered formal neurological evaluation infeasible. Identical inclusion and exclusion criteria were applied across both institutions to ensure inter-cohort comparability. The study protocol was approved by the Institutional Review Boards of both participating centers (approval numbers: KLL-2025-047 and KYLL-2025-160) and conducted in accordance with the Declaration of Helsinki. Individual informed consent was waived given the retrospective nature of data collection; all records were de-identified prior to analysis.

### 2.2 Variable system construction

The candidate variable pool was established through a systematic search of nine Chinese and English databases, including PubMed, Embase, Web of Science, and The Cochrane Library, with no restriction on publication date up to December 2024. Eligible study types encompassed clinical practice guidelines, systematic reviews, meta-analyses, and high-quality prospective and retrospective cohort studies reporting ICU delirium risk factors or prediction models. Methodological quality was appraised using AGREE II for clinical guidelines (14), AMSTAR for systematic reviews and meta-analyses (15), the Newcastle-Ottawa Scale (NOS) for observational studies (16), and PROBAST for prediction model studies (17). A clinical pilot investigation involving 60 consecutive ICU patients was subsequently conducted to evaluate the data completeness, measurement reliability, and clinical feasibility of candidate variables under real-world conditions. Variables with a missing rate exceeding 30%, those subject to systematic measurement bias in mechanically ventilated patients, and those with high inter-variable redundancy were excluded. The final variable system comprised nine baseline characteristics and 24 dynamic variables, the latter constituting the sole input features to the LSTM model.

### 2.3 Time-series data organization

The time origin (T0) was defined as the moment of ICU admission, at which initial dynamic variable values were recorded. Subsequent observation windows were delineated in consecutive 24-hour epochs: T1 (0–24 h), T2 (24–48 h), and so forth, up to a maximum of T7. For each prediction target Tn, the model received as input the complete dynamic variable sequence spanning T0 through T(*n*−1), thereby enabling prospective risk estimation based solely on antecedent clinical data. The maximum observation window was set at 7 days, as 93.6% of delirium events in the internal cohort occurred within this interval; for patients with ICU stays exceeding 7 days, input sequences were truncated at T7, with outcome labels determined by the CAM-ICU assessment status within that window.

Within each 24-hour window, variable-specific aggregation rules were applied: vital signs were summarized as the mean (heart rate, respiratory rate), maximum (body temperature), or minimum (SpO_2_, mean arterial pressure) to capture the most clinically unfavorable physiological state; laboratory values were represented by the most recent measurement within the window; treatment intervention variables (invasive mechanical ventilation, sedative use, antibiotic administration, physical restraint) were encoded as binary indicators of exposure; Richmond Agitation-Sedation Scale (RASS) scores were summarized as the median, and Sequential Organ Failure Assessment (SOFA) scores as the worst value recorded within the window.

### 2.4 Data preprocessing

Continuous variables were retained in their original numeric form and subsequently standardized using *Z*-score normalization; standardization parameters were derived exclusively from the training set and applied uniformly to validation, internal test, and external test sets to prevent distributional leakage. Binary treatment variables were encoded as 0/1. Ordinal variables (RASS: −5 to +4; SOFA: 0–24) retained their original rank values to preserve ordinal information. Extreme physiological values were not truncated or winsorized, as such values frequently carry pathophysiological significance in the critical care context and their attenuation would compromise the integrity of the input signal. Missing values were handled via a masking mechanism implemented within the deep learning framework, whereby a binary mask matrix synchronized with the feature matrix instructed the LSTM units to bypass missing entries during both forward propagation and gradient computation, ensuring that inference was grounded exclusively in observed data. As overall missingness across the dynamic variables remained limited, on the order of one tenth of measurements, and was concentrated in laboratory parameters with lower intra-day sampling frequency, this mechanism enabled the model to learn from all available measurements without resorting to imputation. Class imbalance (delirium prevalence approximately 33%) was addressed by assigning inverse-frequency class weights to positive samples during training.

### 2.5 LSTM model architecture and training

The model was implemented in TensorFlow 2.10.0. Because patients contributed observation sequences of differing length, the masking mechanism additionally operated at the sequence level, flagging windows without available data so that variable-length sequences could be processed without imputing absent time steps. The network architecture comprised two stacked bidirectional LSTM layers, adopted to enable the model to capture both the cumulative trajectory from early admission and the most proximal clinical state within the historical sequence, with a dropout rate of 0.3 applied to each layer to mitigate overfitting, followed by a fully connected output layer with sigmoid activation to yield delirium occurrence probability. The model was trained using binary cross-entropy loss with the Adam optimizer (initial learning rate: 1 × 10⁻^3^) and a batch size of 12. An early stopping callback monitored validation set AUCROC and terminated training if no improvement was observed over six consecutive epochs, at which point the optimal model weights were restored. All stochastic operations were seeded for reproducibility.

### 2.6 Model evaluation and SHAP interpretability analysis

Discrimination was assessed by AUCROC, with 95% CIs estimated via 1,000-iteration bootstrap resampling. The optimal decision threshold was determined by maximizing the Youden index (sensitivity + specificity minus 1) on the internal test set. Supplementary classification metrics including sensitivity, specificity, precision, *F*1 score, and accuracy were computed at this threshold. Calibration was evaluated graphically via calibration curves; clinical utility was assessed through decision curve analysis (DCA), with the net benefit of model-guided intervention compared against the default strategies of treating all or no patients.

SHAP-based interpretability analysis was performed using the GradientExplainer algorithm from the SHAP library (version 0.41.0), which estimates feature attributions via integrated gradients. For each observation window T1–T7, mean absolute SHAP values were computed across all samples to quantify the population-level contribution of each dynamic variable. Analyses were focused on the ten highest-contributing features, as attributions beyond this rank approached zero and exerted negligible influence on model decisions. Temporal trajectories of mean absolute SHAP values were visualized as line plots and feature-window heatmaps across T1–T7. Per-window SHAP summary plots were generated to characterize the directionality of each feature's contribution to predicted delirium risk.

## 3 Results

### 3.1 Patient characteristics

A total of 464 patients were enrolled in the internal cohort, of whom 156 (33.62%) developed delirium during their ICU stay. The external test set comprised 70 patients, with a delirium incidence of 31.43% (*n* = 22). Baseline characteristics were well balanced between delirium and non-delirium groups in both cohorts. In the internal cohort, delirium patients were significantly older than non-delirium patients (64.50 ± 15.88 vs. 59.97 ± 17.74 years, *P* < 0.01), and exhibited a higher prevalence of cerebrovascular disease (21.15% vs. 11.69%, *P* < 0.05); no statistically significant differences were observed in sex, smoking history, alcohol consumption, or comorbidity burden including hypertension, diabetes mellitus, and chronic pulmonary disease (all *P* > 0.05). In the external test set, no baseline characteristic differed significantly between groups (all *P* > 0.05).

Stratified random sampling yielded a training set (*n* = 324), validation set (*n* = 70), and internal test set (*n* = 70), with delirium incidence rates of 33.64%, 34.29%, and 32.86%, respectively. No statistically significant differences were identified across the three subsets in any of the nine baseline characteristics (all *P* > 0.05), confirming the homogeneity of the modeling dataset (Table 1).

**Table 1:** Baseline balance comparison of the modeling datasets (training set, validation set, and internal test set).

Variable	Training set (*n* = 324)	Validation set (*n* = 70)	Internal test set (*n* = 70)	Statistic	*P* value
Delirium incidence, *n* (%)	109 (33.64)	24 (34.29)	23 (32.86)	*χ*^2^ = 0.05	0.98
Age, years, mean ± SD	61.52 ± 17.15	61.38 ± 17.42	61.46 ± 17.65	*F* = 0.00	1.00
Sex, male, *n* (%)	197 (60.80)	42 (60.00)	43 (61.43)	*χ*^2^ = 0.05	0.97
Smoking history, *n* (%)	39 (12.04)	8 (11.43)	9 (12.86)	*χ*^2^ = 0.10	0.95
Alcohol consumption, *n* (%)	63 (19.44)	14 (20.00)	13 (18.57)	*χ*^2^ = 0.08	0.96
Hypertension, *n* (%)	123 (37.96)	26 (37.14)	27 (38.57)	*χ*^2^ = 0.05	0.97
Diabetes mellitus, *n* (%)	102 (31.48)	22 (31.43)	22 (31.43)	*χ*^2^ = 0.00	1.00
Chronic pulmonary disease, *n* (%)	121 (37.35)	26 (37.14)	26 (37.14)	*χ*^2^ = 0.00	1.00
Cerebrovascular disease, *n* (%)	48 (14.81)	11 (15.71)	10 (14.29)	*χ*^2^ = 0.09	0.96
Primary diagnosis, *n* (%)				*χ*^2^ = 0.06	1.00
Trauma-related	50 (15.43)	11 (15.71)	11 (15.71)		
Surgical	40 (12.35)	8 (11.43)	9 (12.86)		
Medical	234 (72.22)	51 (72.86)	50 (71.43)		

Continuous variables conforming to normal distribution are expressed as mean ± standard deviation (mean ± SD); group comparisons were performed using one-way analysis of variance. Categorical variables are expressed as frequency (percentage); group comparisons were performed using chi-square test.

Across the internal cohort, a total of 3,241 valid observation windows were collected from 464 patients. The median monitoring duration was significantly shorter in the delirium group than in the non-delirium group [3 (IQR: 2, 5) vs. 8 (IQR: 5, 12) days, *P* < 0.01], with the distribution of monitoring duration differing markedly between groups (*P* < 0.01), consistent with the clinical pattern of early-onset delirium in critically ill patients (Table 2). Overall missing rate across the 24 dynamic variables was 12.37%, with 70.80% of variables exhibiting missing rates below 5%.

**Table 2:** Comparison of dynamic variable monitoring duration between delirium and non-delirium groups.

Indicators	Total (*n* = 464)	Non-delirium (*n* = 308)	Delirium (*n* = 156)	Statistic	*P* value
Duration, days, *M* (Q1, Q3)	6 (3, 10)	8 (5, 12)	3 (2, 5)	*Z* = 16.02	<0.01
Duration distribution, *n* (%)				*χ*^2^ = 71.85	<0.01
1–2 days	81 (17.46)	28 (9.09)	53 (33.97)		
3–5 days	140 (30.17)	82 (26.62)	58 (37.18)		
6–7 days	139 (29.96)	104 (33.77)	35 (22.44)		
≥8 days	104 (22.41)	94 (30.52)	10 (6.41)		

Continuous variables are presented according to normality test results: normally distributed variables are expressed as mean ± standard deviation (*x¯* ± SD); non-normally distributed variables are expressed as median (interquartile range) [*M* (Q1, Q3)]. Non-parametric tests (Mann–Whitney *U* test) were applied for between-group comparisons that did not meet the assumption of normality.

### 3.2 Internal test set performance

**Figure 1:**
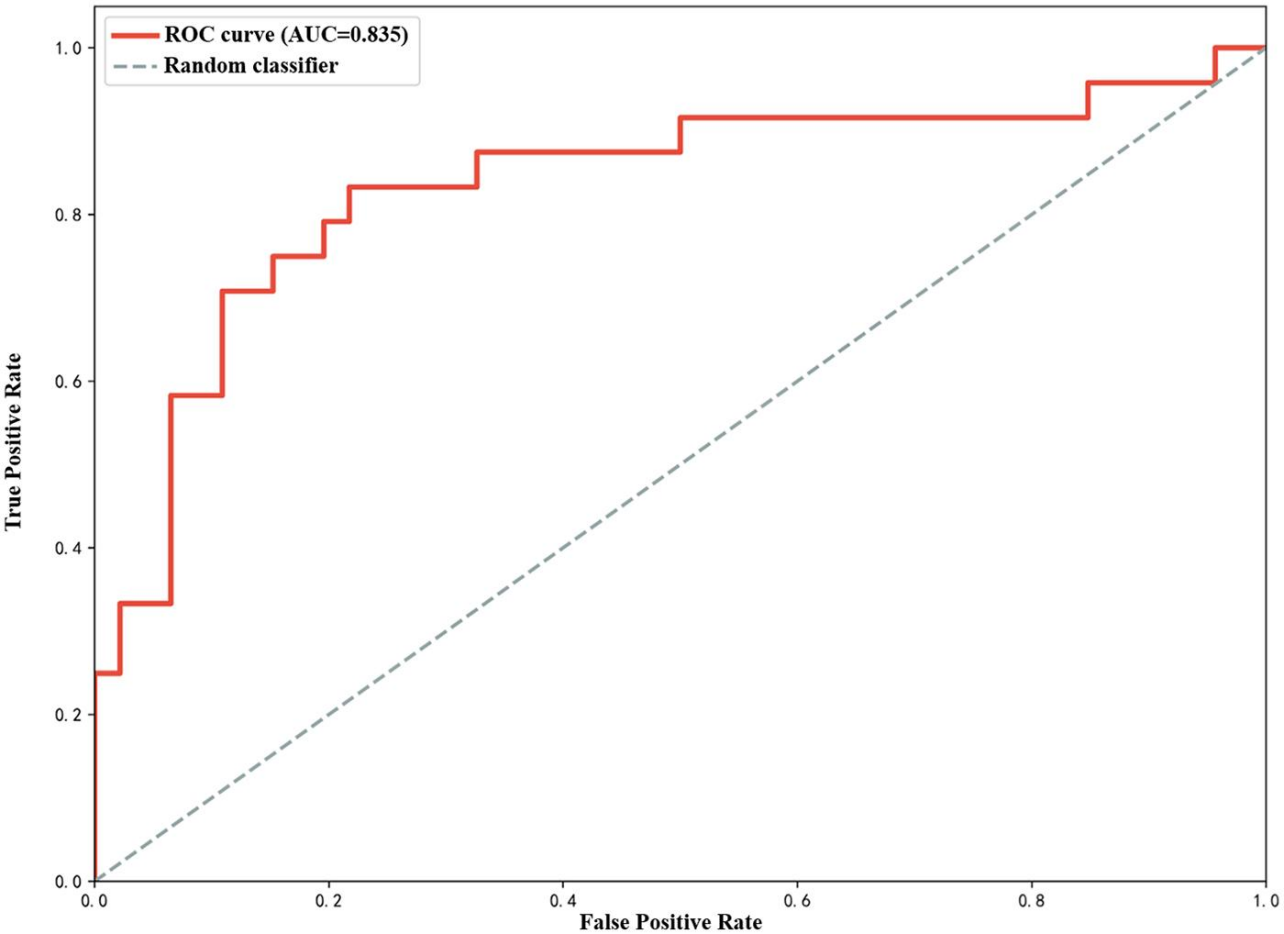
Receiver operating characteristic (ROC) curve of the LSTM model on the internal test set. The area under the ROC curve (AUCROC) was 0.835 (95% CI: 0.724–0.946). The curve demonstrates a rapid ascent toward the upper-left quadrant, indicating effective discrimination at low false-positive rates.

**Figure 2:**
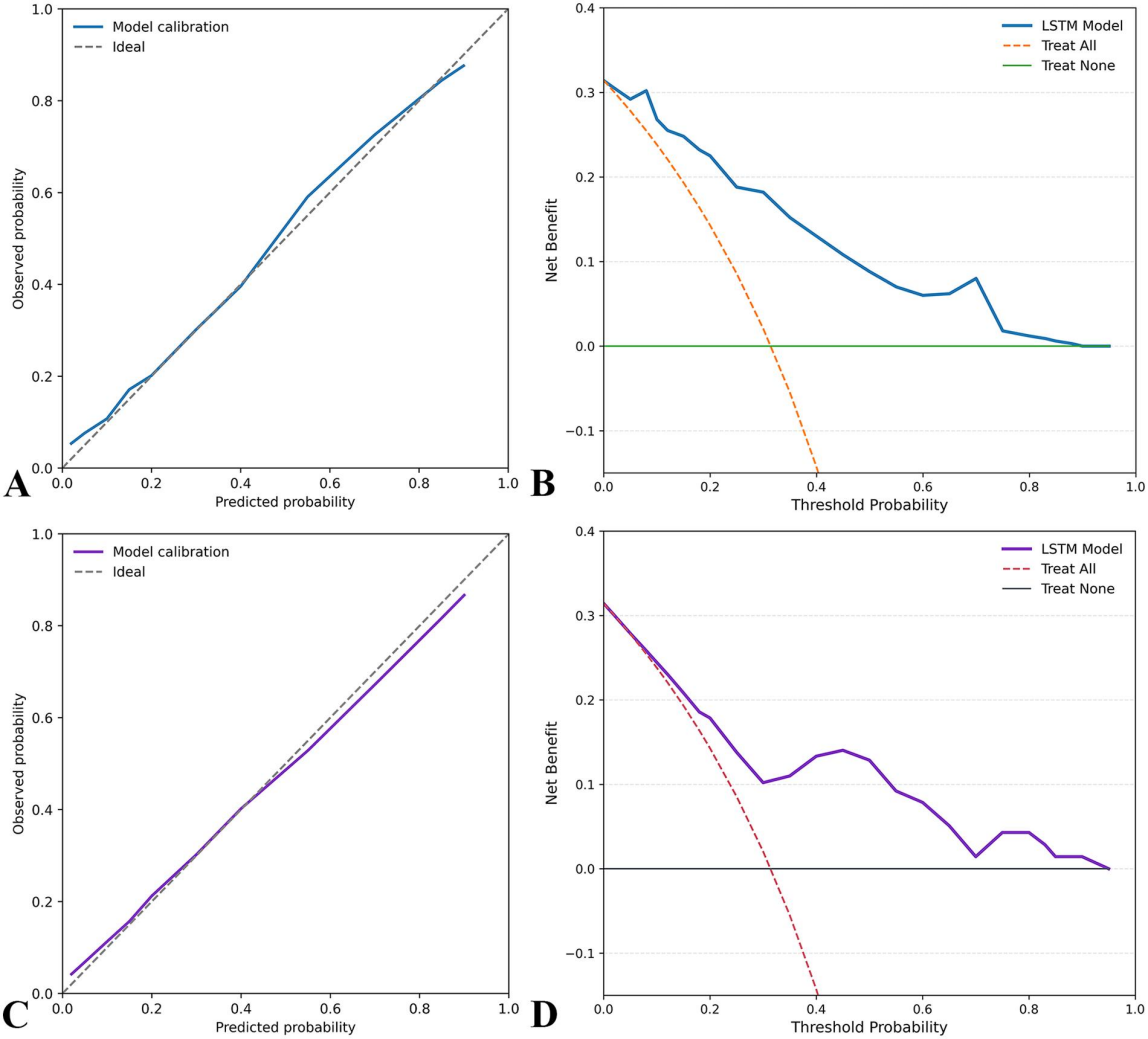
Calibration curves and decision curve analyses (DCA) of the LSTM model in the internal and external test sets. **(A)** Calibration curve in the internal test set; the curve approximates the 45° diagonal reference line, indicating satisfactory agreement between predicted probabilities and observed delirium event rates. **(B)** DCA in the internal test set; the model provides net benefit over the treat-all and treat-none reference strategies across a probability threshold range of approximately 5%–70%. **(C)** Calibration curve in the external test set; the curve lies close to the 45° reference line, indicating that the model's probability outputs remain acceptably reliable in the external cohort. **(D)** DCA in the external test set; the model provides net benefit over the treat-all and treat-none strategies across a probability threshold range of approximately 5%–65%, with smaller net benefit at higher thresholds.

On the internal test set (*n* = 70), the LSTM model achieved an AUCROC of 0.835 (95% CI: 0.724–0.946), with the ROC curve demonstrating a rapid ascent toward the upper-left quadrant, indicating effective discrimination at low false-positive rates (Figure 1). Sensitivity was 0.696 (95% CI: 0.478–0.870), specificity 0.894 (95% CI: 0.787–0.957), precision 0.762 (95% CI: 0.571–0.909), *F*1 score 0.727 (95% CI: 0.561–0.867), and overall accuracy 0.829 (95% CI: 0.729–0.914). The calibration curve demonstrated satisfactory concordance between predicted probabilities and observed event rates, with no evidence of systematic over- or under-prediction. DCA confirmed that the model conferred net clinical benefit over both the treat-all and treat-none reference strategies across a probability threshold range of approximately 5%–70% (Figures 2A,B).

### 3.3 External validation

Comparability between the internal cohort and external test set was confirmed across all nine baseline variables, including delirium incidence (*P* = 0.76), age, sex distribution, comorbidity prevalence, and primary diagnosis category (all *P* > 0.05), establishing an appropriate foundation for cross-institutional generalizability assessment (Table 3).

**Table 3:** Comparison of baseline characteristics between internal and external cohorts.

Variable	Internal cohort (*n* = 464)	External test set (*n* = 70)	Statistic	*P* value
Delirium incidence, *n* (%)	156 (33.62)	22 (31.43)	*χ*^2^ = 0.10	0.76
Age, years, mean ± SD	61.49 ± 17.26	61.07 ± 13.33	*t* = 0.19	0.85
Sex, male, *n* (%)	282 (60.78)	42 (60.00)	*χ*^2^ = 0.01	0.91
Smoking history, *n* (%)	56 (12.07)	8 (11.43)	*χ*^2^ = 0.02	0.90
Alcohol consumption, *n* (%)	90 (19.40)	15 (21.43)	*χ*^2^ = 0.12	0.73
Hypertension, *n* (%)	176 (37.93)	23 (32.86)	*χ*^2^ = 0.50	0.48
Diabetes mellitus, *n* (%)	146 (31.47)	31 (44.29)	*χ*^2^ = 3.42	0.07
Chronic pulmonary disease, *n* (%)	173 (37.28)	21 (30.00)	*χ*^2^ = 1.02	0.31
Cerebrovascular disease, *n* (%)	69 (14.87)	9 (12.86)	*χ*^2^ = 0.15	0.70
Primary diagnosis, *n* (%)			*χ*^2^ = 0.52	0.77
Trauma-related	72 (15.52)	7 (10.00)		
Surgical	57 (12.28)	12 (17.14)		
Medical	335 (72.20)	51 (72.86)		

Continuous variables conforming to normal distribution are expressed as mean ± standard deviation (mean ± SD); group comparisons were performed using independent samples *t*-test. Categorical variables are expressed as frequency (percentage); group comparisons were performed using chi-square test. Fisher's exact test was applied when the minimum expected frequency was less than 5.

**Figure 3:**
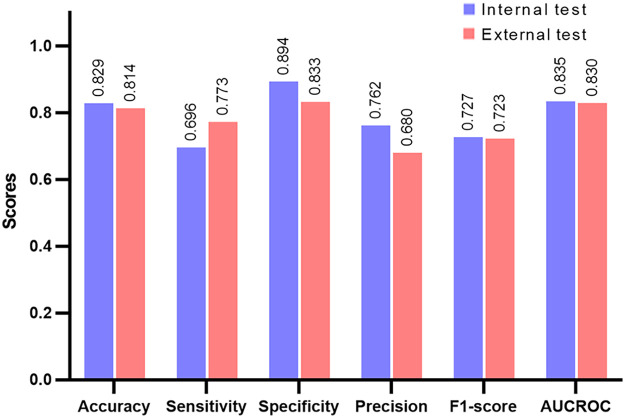
Comparison of model performance metrics between the internal test set and the external test set. AUCROC, area under the receiver operating characteristic curve; CI, confidence interval.

On the external test set (*n* = 70), the model yielded an AUCROC of 0.830 (95% CI: 0.716–0.944), representing a decrement of merely 0.005 relative to internal test set performance, with substantially overlapping confidence intervals between the two estimates. Sensitivity on the external test set was 0.773 (95% CI: 0.545–0.913), specificity 0.833 (95% CI: 0.708–0.921), precision 0.680 (95% CI: 0.480–0.848), *F*1 score 0.723 (95% CI: 0.550–0.868), and accuracy 0.814 (95% CI: 0.714–0.900). The marginal shift in the sensitivity-specificity balance relative to internal performance, characterized by improved sensitivity and modestly reduced specificity, reflects a slight recalibration of the decision boundary in the external institutional context, which can be addressed through threshold optimization tailored to site-specific delirium prevalence. Calibration and decision curve analyses were additionally performed in the external test set. The external calibration curve lay close to the 45° reference line, indicating acceptably reliable probability outputs, and decision curve analysis showed net benefit over the treat-all and treat-none strategies across the principal threshold range of approximately 5%–65%, with limited benefit at higher thresholds (Figures 2C,D). The overall discriminative stability across institutions is illustrated in Figure 3.

### 3.4 SHAP temporal analysis

**Figure 4:**
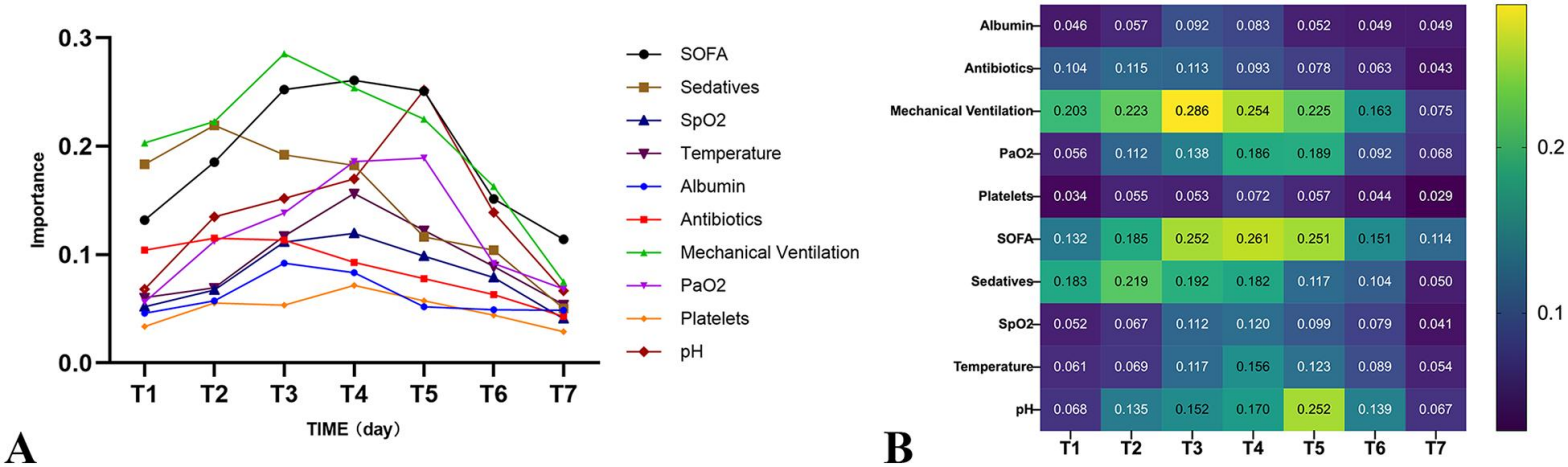
Temporal evolution of SHAP feature importance across observation windows T1–T7. **(A)** Line plot illustrating the dynamic trajectories of mean absolute SHAP values for the top 10 contributing features across successive observation windows. **(B)** Heatmap presenting mean absolute SHAP values across all feature-window combinations; brighter shading indicates higher feature attribution.

SHAP-based interpretability analysis of the internal test set revealed a clinically coherent, phase-dependent transition in the dominant predictors of delirium risk across the T1–T7 observation windows (Figures 4A,B).

During the early phase (T1–T3), iatrogenic intervention variables constituted the primary model drivers. Invasive mechanical ventilation exhibited the highest feature attribution throughout this period, reaching peak mean |SHAP| of 0.286 at T3. Sedative exposure maintained consistently elevated attribution across T1–T4, with a maximum mean |SHAP| of 0.219. These findings are consistent with the established pathophysiological role of invasive ventilation and cumulative sedative burden in precipitating early-onset ICU delirium.

During the intermediate phase (T4–T6), the relative contribution of iatrogenic variables declined, while internal milieu indicators assumed dominance. Arterial pH emerged as the principal predictor at T5, attaining a mean |SHAP| of 0.252 and surpassing the concurrent contribution of invasive mechanical ventilation. SOFA score rose in parallel, peaking at a mean |SHAP| of 0.261 at T4 and remaining elevated at 0.251 at T5, jointly constituting the core decision basis during this phase. Body temperature and PaO_2_ demonstrated stable intermediate-level attributions throughout T4–T6.

During the late phase (T7), absolute SHAP values declined across all features; however, SOFA score retained the highest relative attribution within this window (mean |SHAP| = 0.114), suggesting a progressively prominent role of multiorgan dysfunction in delirium risk as ICU stay extended. Interpretation of T7 findings warrants caution given the limited sample availability at this observation window. As shown in Table 2, only 6.41% of delirium patients maintained monitoring durations of 8 days or more, which substantially constrains the sample size available for T7 analysis.

**Figure 5:**
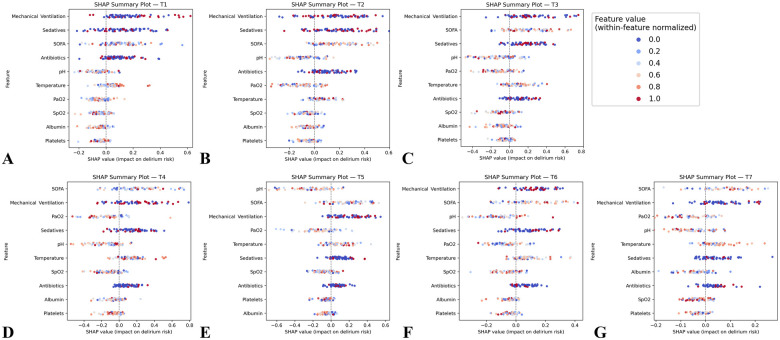
Per-window SHAP summary plots at observation windows T1–T7. Each plot displays the distribution of individual SHAP values for the top 10 features, with feature values encoded by color; positive SHAP values indicate promotion of delirium risk and negative values indicate suppression. Panels (**A–G**) correspond to observation windows T1–T7, respectively.

Per-window SHAP summary plots revealed three distinct attribution patterns in the directionality of feature contributions (Figure 5). SOFA score and body temperature exhibited positive-direction driving patterns, whereby higher values corresponded consistently with positive SHAP values, reflecting direct upward pressure on predicted delirium probability. Arterial pH and PaO_2_ demonstrated inverse-direction patterns, wherein lower values were associated with positive SHAP attributions, indicating that acid-base dysregulation and impaired oxygenation were associated with higher predicted delirium risk. Invasive mechanical ventilation and sedative use exhibited complex intervention-attribute patterns, with exposure states corresponding predominantly to positive SHAP values, which may reflect both the greater severity of underlying illness in patients requiring such interventions and a potential independent association between these iatrogenic exposures and delirium risk.

## 4 Discussion

### 4.1 Model performance and the value of temporal modeling

The bidirectional LSTM model achieved comparable AUCROC values on the internal and external test sets, with a cross-institutional decrement of only 0.005 and substantially overlapping confidence intervals. This degree of generalizability compares favorably with existing prediction models for ICU delirium. Gong et al. reported an AUCROC of 0.845 using a multi-source machine learning framework applied to large-scale public databases (18). However, their study benefited from substantially larger training samples and lacked a prospectively defined external validation cohort from an independent institution. Contreras et al. achieved an AUCROC of 0.870 with a CatBoost-based dynamic prediction approach, but no independent external validation was performed (19). In contrast, the random forest model developed by Hur et al. achieved an AUCROC of 0.92 internally but degraded to 0.721 upon external validation against the MIMIC-III database (20), a pattern frequently observed in single-center models that overfit to institution-specific data distributions. We attribute the minimal performance attenuation in our model to the temporal modeling paradigm of LSTM, which learns generalizable patterns of disease trajectory evolution rather than institution-specific static feature distributions, thereby reducing dependency on site-specific data characteristics.

Despite these favorable discrimination metrics, the internal test set sensitivity of 0.696 should be noted. Approximately 30% of delirium episodes were not identified at the predefined threshold, a limitation with direct clinical implications in the critical care setting. This operating point corresponds to the Youden index, which balances sensitivity and specificity rather than maximizing case detection. Because the model outputs a continuous risk probability, the decision threshold can be lowered in clinical deployment to prioritize sensitivity, accepting more false positives in exchange for fewer missed cases; this trade-off is reasonable in delirium prevention, where the measures prompted by a positive screen are themselves low-risk, and the threshold can be calibrated to the delirium prevalence of the implementing institution. Beyond threshold selection, this sensitivity ceiling likely reflects, in part, the 24-hour observation window adopted in this study. Although this window was necessitated by the limited intra-day sampling frequency of key laboratory parameters identified during the pilot investigation, it yields coarser temporal resolution than the 12-hour windows employed by Gong et al. (18) and Contreras et al. (19). Additionally, we excluded neurophysiological monitoring data, including electroencephalography and sleep quality indices, from the variable system because of their high missing rates and insufficient standardization in the participating ICUs. This exclusion may have limited the model's capacity to detect delirium-associated neural perturbations before clinical manifestation.

The pathophysiological trajectory of ICU delirium is inherently dynamic: physiological parameters fluctuate at high frequency, therapeutic effects accumulate over time, and the interplay between metabolic derangement and organ dysfunction evolves continuously. Static prediction models compress these multi-temporal data into a single feature vector, discarding temporal ordering and the evolutionary dynamics of the clinical signal (21, 22). The LSTM architecture addresses this limitation through gating mechanisms that selectively retain clinically meaningful long-range dependencies while attenuating transient fluctuations (9), enabling the model to capture cumulative and interactive effects across the full observation sequence. The SHAP findings from this study provide indirect empirical support for the value of temporal modeling. The phase-dependent transition in dominant predictors across T1 through T7 demonstrates that risk-driving factors undergo systematic reorganization as the clinical course progresses. A static model cannot distinguish between the early iatrogenic-dominant and later internal milieu-dominant risk profiles, and would instead produce a temporally averaged attribution that misrepresents the actual risk architecture at any given time point. A fair comparison with simpler static models is difficult to construct within this dataset, as reducing the dynamic sequences to the fixed-length feature vector such models require would discard the temporal evolution in which much of the delirium-related signal resides and confound modeling paradigm with information availability. The architecture therefore reflects the demands of variable-length, multivariate, and partially missing time-series data rather than a preference for complexity.

### 4.2 Clinical implications of temporal feature attribution

The SHAP analysis revealed a clinically coherent, three-phase transition in the dominant drivers of delirium risk, with direct implications for the timing and focus of preventive interventions. During the early phase (T1–T3), invasive mechanical ventilation and sedative exposure emerged as the primary contributors. This finding aligns with established pathophysiological mechanisms: mechanical ventilation induces somatic stress responses and is accompanied by sedative administration, which suppresses central nervous system activity, disrupts circadian rhythm, and impairs normal sleep architecture. These results lend quantitative support to the early liberation and sedation minimization strategies endorsed by the PADIS clinical practice guidelines (23), including daily spontaneous awakening trials, early extubation protocols, and sedation titration to the lightest effective depth.

During the intermediate phase (T4–T6), arterial pH and SOFA score emerged as dominant predictors, reflecting a transition from acute iatrogenic stress to progressive deterioration of the internal milieu. Metabolic acidosis has been implicated in delirium pathogenesis through activation of neuroinflammatory pathways and increased blood-brain barrier permeability, which facilitates the entry of inflammatory mediators into the central nervous system (24). The concurrent rise in SOFA score attribution suggests that multiorgan dysfunction begins to exert independent pressure on delirium risk during this phase.

During the late phase (T7), SOFA score remained the most influential predictor, reflecting the increasing centrality of multiorgan dysfunction in delirium risk. This finding is consistent with established mechanisms by which systemic inflammatory mediators and accumulated metabolic waste products exert neurotoxic effects through a compromised blood-brain barrier (25, 26). Importantly, SHAP values quantify statistical associations between input features and model output rather than causal effects.

The dynamic structure of the model also lends itself to direct integration into clinical workflow. Because risk is re-estimated within each 24-hour window from all preceding data, the model can be embedded within the electronic health record to generate continuously updated, patient-level risk estimates that flag high-risk patients in real time and direct clinical attention toward the phase-appropriate predictors identified above. Operating at a threshold favoring sensitivity, such a system would function as an automated screen that prompts pre-emptive, low-risk preventive measures before delirium becomes clinically manifest, complementing rather than replacing periodic bedside assessment. Realized in this form, the model would shift delirium surveillance from intermittent manual screening toward continuous, anticipatory risk monitoring, an effect whose impact on delirium incidence and patient outcomes warrants evaluation in prospective implementation studies.

### 4.3 Limitations

This study has several limitations. First, the retrospective design introduces the possibility of selection bias and information bias, with data quality constrained by the completeness and standardization of electronic health record documentation at the participating institutions; moreover, the sample size remains modest for deep learning, which may limit the precision and reproducibility of the reported performance estimates. Second, external validation was restricted to a single institution within the same metropolitan area and was based on a relatively small external cohort, limiting the generalizability assessment; multicenter validation across geographically diverse settings is required to establish broader applicability. Third, a static comparator model was not developed within the same dataset, precluding direct quantification of the incremental predictive value of temporal sequence modeling over single-time-point approaches. Fourth, the maximum observation window of 7 days leaves model performance in patients with extended ICU stays uncharacterized. Fifth, this study represents a retrospective validation only. Prospective evaluation through integration of the model into clinical information systems and assessment of its impact on delirium incidence and patient outcomes remains an essential step toward clinical translation (27, 28).

### 4.4 Future directions

Several avenues warrant exploration in future research. First, multicenter validation across geographically and institutionally diverse settings would more rigorously evaluate the generalizability of the model. Second, shortening the observation window from 24 to 12 h may improve temporal resolution and enhance sensitivity for detecting early delirium signals, provided that sufficient laboratory data can be obtained at this frequency. Third, incorporating neurophysiological monitoring data, such as electroencephalography patterns and sleep quality indices, may capture neural perturbations that precede clinical manifestation of delirium, thereby improving presymptomatic detection. Fourth, developing and benchmarking a static comparator model within the same dataset would enable direct quantification of the incremental value conferred by temporal sequence modeling. Finally, prospective integration of the model into bedside clinical decision support systems, coupled with assessment of its impact on delirium prevention rates and patient outcomes, represents the critical next step toward clinical translation.

## 5 Conclusion

The bidirectional LSTM model demonstrated stable and generalizable discriminative performance for dynamic ICU delirium prediction across internal and external validation. SHAP-derived temporal attribution revealed a phase-specific transition in dominant risk factors across the ICU stay, offering an evidence-based rationale for stage-differentiated clinical monitoring and intervention strategies. These findings suggest that combining temporal deep learning with interpretability analysis can advance the early identification and targeted prevention of ICU delirium. Prospective validation within real-time clinical decision support systems represents the essential next step toward clinical translation.

## Data Availability

The original contributions presented in the study are included in the article/Supplementary Material, further inquiries can be directed to the corresponding author.
